# Preparation and Supercooling Modification of Salt Hydrate Phase Change Materials Based on CaCl_2_·2H_2_O/CaCl_2_

**DOI:** 10.3390/ma10070691

**Published:** 2017-06-23

**Authors:** Xiaoxiao Xu, Zhijun Dong, Shazim Ali Memon, Xiaohua Bao, Hongzhi Cui

**Affiliations:** 1 College of Civil Engineering, Shenzhen University, Shenzhen 518060, China; 2150150414@email.szu.edu.cn (X.X.); bxh@szu.edu.cn (X.B.); 2 School of Traffic and Environment, Shenzhen Institute of Information Technology, Shenzhen 518060, China; dongzj@sziit.edu.cn; 3 Department of Civil Engineering, School of Engineering, Nazarbayev University, Astana 010000, Kazakhstan; shazim.memon@nu.edu.kz

**Keywords:** phase change materials, calcium chloride hexahydrate, supercooling, Nano-SiO_2_

## Abstract

Salt hydrates have issues of supercooling when they are utilized as phase change materials (PCMs). In this research, a new method was adopted to prepare a salt hydrate PCM (based on a mixture of calcium chloride dihydrate and calcium chloride anhydrous) as a novel PCM system to reduce the supercooling phenomenon existing in CaCl_2_·6H_2_O. Six samples with different compositions of CaCl_2_ were prepared. The relationship between the performance and the proportion of calcium chloride dihydrate (CaCl_2_·2H_2_O) and calcium chloride anhydrous (CaCl_2_) was also investigated. The supercooling degree of the final PCM reduced with the increase in volume of CaCl_2_·2H_2_O during its preparation. The PCM obtained with 66.21 wt % CaCl_2_·2H_2_O reduced the supercooling degree by about 96.8%. All six samples, whose ratio of CaCl_2_·2H_2_O to (CaCl_2_ plus CaCl_2_·2H_2_O) was 0%, 34.03%, 53.82%, 76.56%, 90.74%, and 100% respectively, showed relatively higher enthalpy (greater than 155.29 J/g), and have the possibility to be applied in buildings for thermal energy storage purposes. Hence, CaCl_2_·2H_2_O plays an important role in reducing supercooling and it can be helpful in adjusting the solidification enthalpy. Thereafter, the influence of adding different percentages of Nano-SiO_2_ (0.1 wt %, 0.3 wt %, 0.5 wt %) in reducing the supercooling degree of some PCM samples was investigated. The test results showed that the supercooling of the salt hydrate PCM in Samples 6 and 5 reduced to 0.2 °C and 0.4 °C respectively. Finally, the effect of the different cooling conditions, including frozen storage (−20 °C) and cold storage (5 °C), that were used to prepare the salt hydrate PCM was considered. It was found that both cooling conditions are effective in reducing the supercooling degree of the salt hydrate PCM. With the synergistic action of the two materials, the performance and properties of the newly developed PCM systems were better especially in terms of reducing the supercooling degree of the PCM. The novel composite PCMs are promising candidates for thermal energy storage applications.

## 1. Introduction

Phase change materials (PCMs), which are widely being used in many fields including buildings and solar applications, have attracted the research community due to their high energy storage and small temperature change from storage to retrieval [1,2]. In comparison to organic PCMs, inorganic PCMs such as salt hydrates are more preferred due to their high latent storage, non-flammability, good thermal conductivity, and low cost [3]. However, many salt hydrate PCMs undergo serious supercooling and phase decomposition during the process of releasing latent heat, which limits their utilization in practical applications. Extremely tiny nucleation agents have been used to influence the supercooling of hydrate salt PCMs. These nucleation agents when added to the melted PCM will stimulate crystallization during freezing [4,5,6,7].

The CaCl_2_·6H_2_O PCM has high latent heat, a low phase-transition temperature (29.5 °C), and is more suitable for utilization in building for energy conservation or power saving in summer [8]. It is a by-product (waste) commonly obtained from many chemical processes such as the production of soda ash. The formation of CaCl_2_·2H_2_O usually depends on water loss from CaCl_2_·6H_2_O by heating it to 175.5 °C. In addition, it is convenient to develop CaCl_2_·2H_2_O from CaCl_2_·6H_2_O. However, high purity CaCl_2_·6H_2_O has serious supercooling during freezing, which restricts its practical application as a phase change material and needs to be avoided. Lane [4] introduced different kinds of inorganic salt (as additive) having a similar crystal structure to that of CaCl_2_·6H_2_O to reduce the supercooling degree of CaCl_2_·6H_2_O. During the past decades, investigators have provided some explanation, reasons, and instructions for further research. Fellchenfeld and Sarig [9] hold a view that a specific nucleation agent should have a structure similar to that of the crystal it is going to nucleate. While investigating CaCl_2_·6H_2_O, the authors concluded that the purpose of a nucleation agent is to ensure that the hexahydrate crystallizes and makes the system more stable. Agron and Busing [10] compared CaCl_2_·6H_2_O with SrCl_2_·6H_2_O and found that the interatomic distances, bond angles, and dihedral angles between planes are all similar. The crystal structure similarity helps liquid CaCl_2_·6H_2_O easily adhere to the surface of SrCl_2_·6H_2_O and to crystallize when the temperature of the liquid falls below the phase-transition point. Hence, the researchers commonly utilized inorganic salts or some hydrate salts with the goal to reduce the supercooling of PCMs, and effective results were obtained.

Nano additives have been used to eliminate the supercooling of different PCM systems [11,12,13]. It is known that the shape and size of nanoparticles could have positive effects on nucleation during the crystal growth process of a PCM. He et al. [12] used TiO_2_ nanoparticles in a BaCl_2_ aqueous solution, and reduced the supercooling degree of a PCM system by 84.92%. Ma et al. [13] reported that nano-TiO_2_ reduced the supercooling of Al_2_(SO_4_)_3_·18H_2_O by 88.39%. Metal nanoparticles, metallic oxide nanoparticles, and metal nitride nanoparticles (Cu, Al_2_O_3_, and AlN) added into a PCM can also stimulate and ensure a timely crystallization so as to reduce the supercooling that occurs during solidification [14,15,16]. Zhang et al. [17] dispersed hydrophobic SiO_2_ nanoparticles as nucleating agents into n-Octacosane emulsions, and found that the composite with a 0.3 wt % concentration of SiO_2_ nanoparticles was most effective in eliminating supercooling. Li et al. [18] evaluated the performance of graphene, SiO_2_ nanofluids, and TiO_2_ nanofluids (with different additive volumes) in reducing the supercooling degree of pure water. The authors found that graphene was the most effective, and with a small dosage (0.02 wt % ± 0.001) can entirely eliminate the supercooling degree of pure water. Hence, in recent years, nano materials, which use salt or hydrate salt, are more preferred due to its lower additive volume and higher efficiency.

However, the literature evaluating the performance of CaCl_2_·6H_2_O and similar PCMs based on CaCl_2_ with nano particles is scarce. According to the researchers in Ref. [19], CaCl_2_·6H_2_O is a promising PCM for heating and cooling processes in building applications. However, in their research [19], the data of DSC (Differential Scanning Calorimetry) measurements under different cycles seemed to be random and instable. Some researchers insisted that adding additives can suppress the formation of CaCl_2_·4H_2_O, which leads to the segregation of a PCM [20,21]. Chmit et al. [21] investigated the relationships between the volume of CaCl_2_ and H_2_O for the preparation of CaCl_2_·6H_2_O. They found that the maximum enthalpy was obtained with a 50.66 wt % of CaCl_2_. However, they also found that the formation of CaCl_2_·4H_2_O as well as the sample volume would result in the instability of DSC data.

It is pertinent to mention here that in the existing studies, the authors have seldom discussed the preparation method of CaCl_2_·6H_2_O. Moreover, as per the authors’ knowledge, no researchers have explored the relationships between the additive volumes of CaCl_2_·2H_2_O and CaCl_2_. Hence, in this paper, a novel salt hydrate PCM (CaCl_2_·2H_2_O-CaCl_2_-H_2_O) was prepared to reduce supercooling by evaluating the relationship between the proportion of calcium chloride dihydrate (CaCl_2_·2H_2_O) and calcium chloride anhydrous (CaCl_2_). The role of CaCl_2_·2H_2_O in reducing supercooling as well as its effect on adjusting the solidification enthalpy was investigated. Nano SiO_2_ was selected to modify and eliminate the supercooling of the PCM system to less than a degree. The method applied in this research to prepare the salt hydrate PCM was relatively uncomplicated and easy to use. Finally, the effect of the different cooling conditions, including frozen storage (−20 °C) and cold storage (5 °C), that were used to prepare the salt hydrate PCM was considered.

## 2. Experimental Section

### 2.1. Materials Selection

Calcium chloride dihydrate (CaCl_2_·2H_2_O, purity >99%) and calcium chloride anhydrous (CaCl_2_, purity >99%) were obtained from Guangdong, Huada Chemical Co., Ltd. (Shenzhen, China). The deionized water (H_2_O) was obtained from Foshan Hugke Water Treatment Equipment Co., Ltd. (Foshan, China). The Nano SiO_2_ (SiO_2_, purity >99.5%, diameter: 15 ± 5 nm) was purchased from Shanghai Macklin Biochemical Co., Ltd. (Shanghai, China).

### 2.2. Preparation of PCMs

In this paper, a new method was used to prepare the CaCl_2_·2H_2_O and CaCl_2_ as a salt hydrate PCM. The principle of how a PCM system works is based on the phase-transition mechanism of CaCl_2_·6H_2_O. Based on the molecular formula of CaCl_2_·6H_2_O, we have known that CaCl_2_:6H_2_O = 111:108. In order to prepare the salt hydrate PCM, a degree of supersaturation was required. At first, we prepared a saturated CaCl_2_ solution, which was required for the salt hydrate PCM. Table 1 shows the solubility of CaCl_2_ in 100 g water under different liquid temperatures.

It can be seen that when the temperature of the water is 30 °C, the solubility of CaCl_2_ is 100 g, and is similar to the molecular weight ratio of CaCl_2_ to H_2_O in CaCl_2_·6H_2_O (111:108). In this experiment, we calculated the concentration of CaCl_2_ and CaCl_2_·2H_2_O by using Equation (1).

(1) $$\frac{x+\frac{111}{147}y}{\frac{36}{147}y+z}=1$$

where *x* and *y* represent the weight of CaCl_2_ and CaCl_2_·2H_2_O molecules respectively; and *z* represents the weight of the deionized water which was used as a solvent for the CaCl_2_ and CaCl_2_·2H_2_O.

Equation (1) reflects the mass ratio of CaCl_2_ and water molecules. As the molar mass of CaCl_2_·2H_2_O is 147 g/mol, this means that 147 g of CaCl_2_·2H_2_O contains 36 g of water and 111 g of CaCl_2_. In this research, we used 50 g of deionized water as a solvent. The right hand side of Equation (1) should be equal to 1.03 (111/108 = 1.03). However, during the preparation process, the evaporation of water should be considered. Hence, the ratio of CaCl_2_ to H_2_O in Equation (1) was set to 1. Considering the boundary condition of the equation, the value of parameter *x* can be fixed as 50 g. The details of the experimental matrix used to prepare the salt hydrate PCM with different values of CaCl_2_ (*x*), CaCl_2_·2H_2_O (*y*), and H_2_O (*z*) are listed in Table 2. During production, the ratios of all of the raw materials have to be kept. It is worth mentioning here that the yield of the product produced depends on the scale of production.

For the preparation of samples, different concentrations of CaCl_2_ and CaCl_2_·2H_2_O were weighted and added into deionized water. An agitator (RW 20 D S025, IKA, Guangzhou, China) operated at a 500 r/min speed was employed to mix the materials for 30 min, while a water-bath (HH-2, Zhiborui Instrument Manufacturer, Changzhou, China) was utilized to ensure that the temperature during the experiment remained at 30 °C. After mixing all of the ingredients, the solution was kept in a refrigerating cabinet for about 12 h so as to stimulate crystallization. This method requires fast freezing the solution to reach −20 °C, so that the quality of the salt hydrate PCM can be guaranteed. In order to explore the effect of nano SiO_2_ as a nucleation agent in reducing supercooling, an ultrasonic homogenizer (JY-92-IIN, Ningbo Xinzhi Biotech Co., Ltd., Ningbo, China) was used to disperse the nano particles. For this purpose, firstly, the nano SiO_2_ particles were dispersed in deionized water, which was followed by the mixing of the CaCl_2_·2H_2_O and the CaCl_2_ in the nanofluid. This technique is more convenient and effective than directly adding nano particles into the melted PCM. According to this method, nanofluid can easily be mixed with other ingredients. The concentration of SiO_2_ was 0.1%, 0.3%, and 0.5% by weight of the PCMs. After determining the most suitable dosage of SiO_2_ in reducing the supercooling degree of the PCM, the effect of different cooling conditions, including frozen storage (−20 °C) and cold storage (5 °C), that were used for preparing the salt hydrate PCM was also considered.

### 2.3. Experimental Methods and Procedure

**Figure 1:**
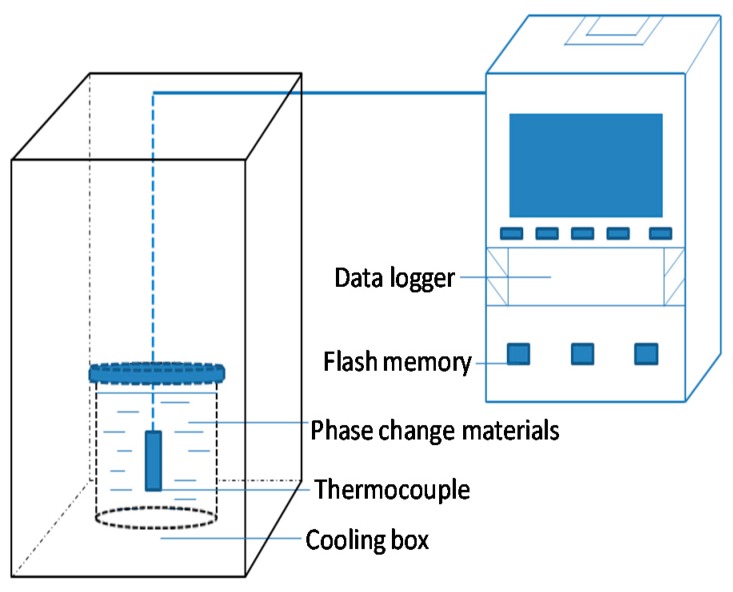
Schematic diagram of the supercooling degree recording system.

In this research, the T-history method was used to record the cooling temperatures of the samples. After preparation, all of the PCM samples were heated in a water bath at 40 °C, followed by keeping the samples in a cooling chamber. The details of the supercooling degree recording system are shown in Figure 1. During the solidification process, the cooling temperature was measured by thermocouples (Type K resolution ±0.3 °C), which were connected to a data recorder (TP700, Shenzhen TOPRIE Electronics Co., Ltd., Shenzhen, China). The temperature of the cooling chamber was set to drop gradually to reach −15 °C.

The mineralogical analysis of samples was carried out by X-ray diffraction (D8 Advanced, Bruker, Karlsruhe, Germany), while the thermal properties including the phase change temperature and the latent heat of the samples were measured by a DSC (Differential Scanning Calorimetry) instrument (DSC-200L, Nanjing Dazhan Electrical Technology Company, Nanjing, China). Finally, to evaluate the dispersion efficiency of the nano particles in the salt hydrate PCM, an environmental scanning electron microscopy (ESEM) (FEI, Quanta TM 250 FEG, Hillsboro, OR, USA) was utilized to capture micrographs of the prepared composite PCM.

## 3. Results and Discussion

### 3.1. Analysis of Mineralogical, Thermal and Supercooling Phenomenon

**Figure 2:**
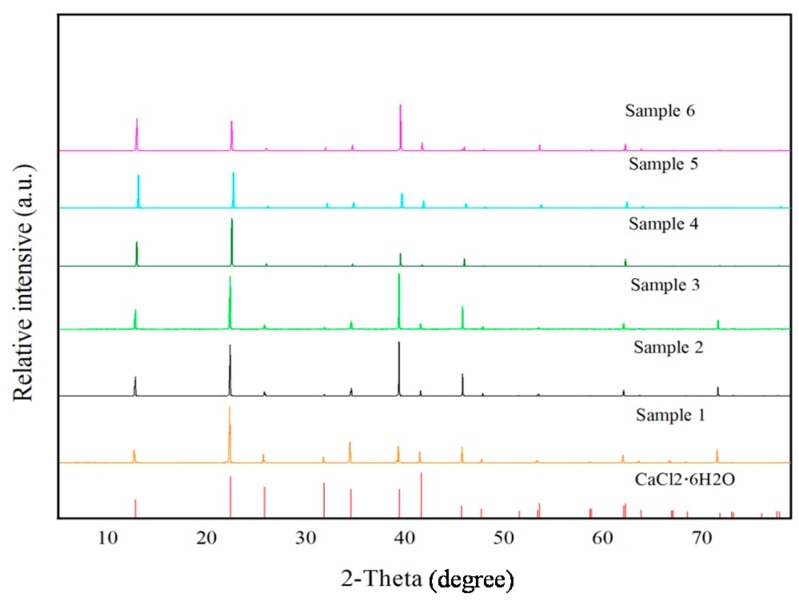
XRD patterns of the prepared six samples and standard CaCl_2_·6H_2_O phase.

**Figure 3:**
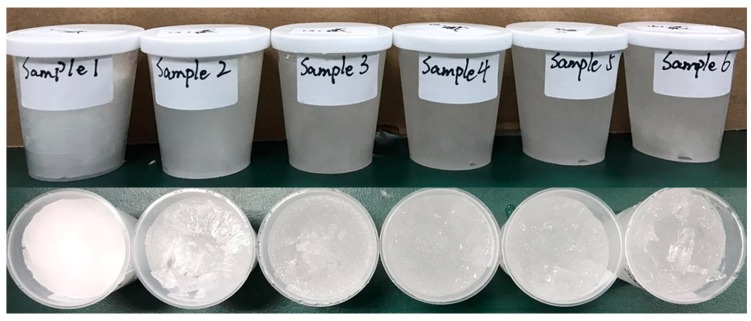
The photo of the prepared six samples containing different volumes of CaCl_2_·2H_2_O and CaCl_2_.

The mineralogical analysis of the prepared samples was carried out by XRD. The XRD spectra of all of the prepared samples are shown in Figure 2. The peak positions reflected are all related to standard CaCl_2_·6H_2_O as provided by the XRD database. Therefore, all of the samples can be considered to follow the phase-transition mechanism of CaCl_2_·6H_2_O. The only difference in spectrum is the height of peak intensity, which is related to the degree of crystallization. It also means that the variation in volume of CaCl_2_·2H_2_O has an influence on the crystallization degree of the salt hydrate PCM. The photographs of all six samples are shown in Figure 3.

**Figure 4:**
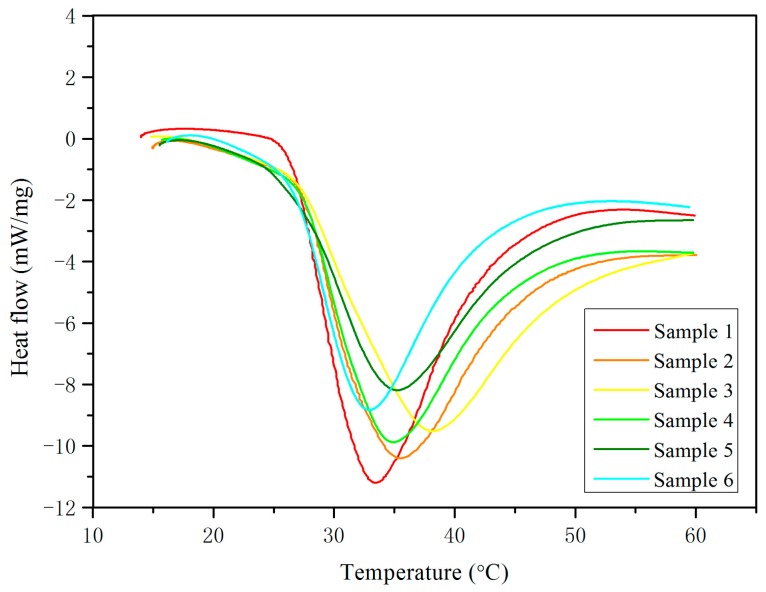
DSC melting curves of Samples 1–6.

The melting curves of all of the samples determined by DSC are shown in Figure 4, while the detailed thermal properties—i.e., the starting phase-transition temperature, the complete melting temperature, and the solidification enthalpy—are presented in Table 3. The solidification enthalpy reduced with the increase in the volume of CaCl_2_·2H_2_O. The reduction in the solidification enthalpy was compromised over improving the supercooling degree of the PCM. The starting phase-transition temperature of all of the samples was found to be around 28 °C. This shows that the prepared PCMs can be used for thermal energy storage applications in buildings, as their phase-transition temperatures are located within the range of the human comfort zone. Among all of the samples, the maximum enthalpy recorded was more than 200 J/g, which is obviously higher than in other research [8]. Sample 6 showed the lowest value of enthalpy (about 155.29 J/g); however, it is still higher than other inorganic PCMs that are used for a building’s energy conservation [7]. Therefore, all six samples have relatively higher enthalpy, and have the possibility to be applied in buildings for thermal energy storage purposes.

**Figure 5:**
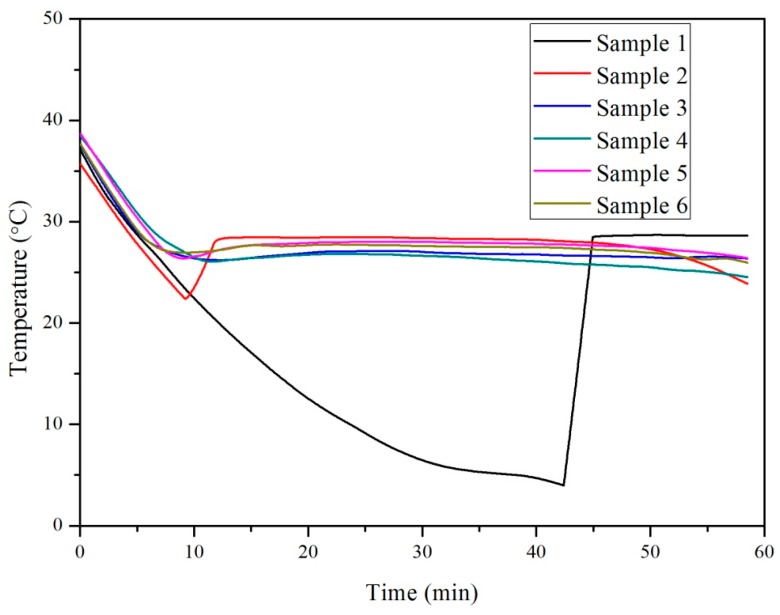
Cooling curve of the six CaCl_2_·2H_2_O-CaCl_2_-H_2_O samples.

The cooling curves of all six samples are shown in Figure 5. The supercooling degree of Samples 1–6 was found to be 24.7 °C, 6.1 °C, 0.9 °C, 0.8 °C, 1.6 °C, and 0.8 °C, respectively. This shows that the supercooling degree reduced with the increase in the volume of CaCl_2_·2H_2_O. Therefore, CaCl_2_·2H_2_O mixed with CaCl_2_ in deionized water can be used to prepare a preferable salt hydrate PCM system. In comparison to Sample 1 without CaCl_2_·2H_2_O, Sample 5 containing a higher volume of CaCl_2_·2H_2_O (58.34 wt %) reduced supercooling to a higher degree. This proves that CaCl_2_·2H_2_O is an effective nucleating agent to reduce the supercooling of salt hydrate PCMs. It can also be seen that the supercooling reduction performance of Sample 2 was not efficient when compared with the other samples. Hence, for the different wt % of CaCl_2_·2H_2_O tried in this research, the minimum volume of CaCl_2_·2H_2_O acceptable for reducing the supercooling of a PCM should be greater than 34.03 wt %.

### 3.2. Influence of Nano-SiO_2_ Addition and Cooling Conditions in Reducing Supercooling

**Figure 6:**
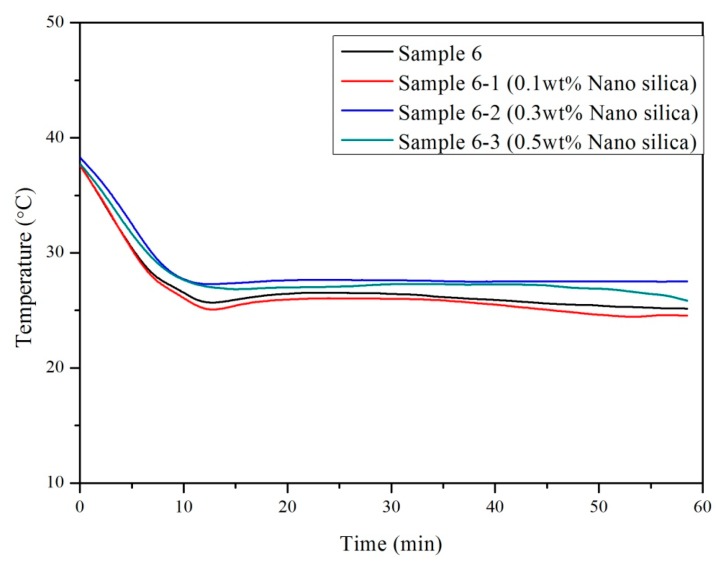
Cooling curve of Sample 6 with different dosages of nano SiO_2_ particles.

After determining the optimum dosage of CaCl_2_·2H_2_O in reducing the supercooling degree of a PCM (Sample 6), the influence of adding different percentages of SiO_2_ in reducing supercooling was determined. For this purpose, Sample 6 was prepared with different dosages of SiO_2_ (0.1%, 0.3%, 0.5% by weight of the PCM). The results of the cooling curves are shown in Figure 6, while the details of the thermal properties are presented in Table 4. In can be seen that the supercooling degree (ΔT = T_m_ − T_n_) reduced with an increase in the percentage of SiO_2_ particles, and it was almost eliminated when the dosage of nano SiO_2_ particles was 0.5 wt %. In comparison to Sample 1 (with only CaCl_2_), the performance of Sample 6 with nano SiO_2_ particles is more promising to be applied as a specific PCM.

**Figure 7:**
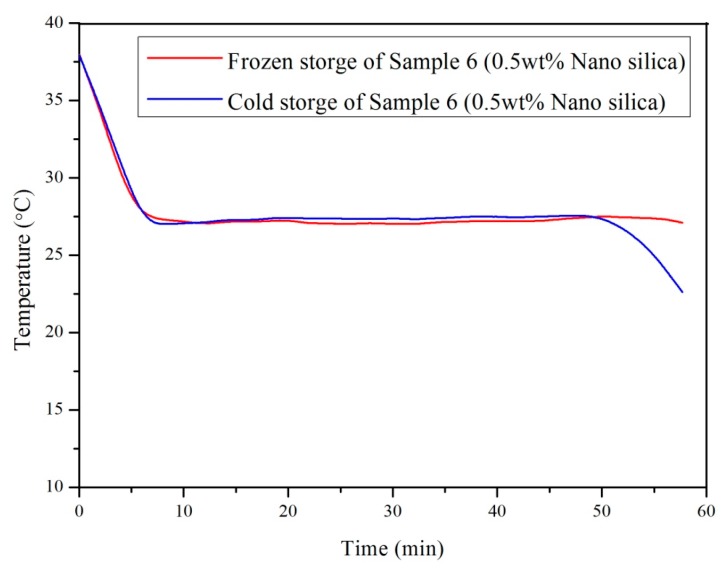
Cooling curve of Sample 6 (0.5 wt % nano SiO_2_ particles) with different cooling conditions.

The cooling curves of Sample 6 (0.5 wt % nano SiO_2_ particles) prepared with different cooling conditions (frozen storage (−20 °C) and cold storage (5 °C)) are compared in Figure 7. For both samples, the supercooling degree was eliminated, and hence the influence of different cooling conditions for preparing the samples can be neglected. However, the storage temperature of PCMs is required to be lower than 5°C to guarantee a better crystallization for the prepared PCM.

**Figure 8:**
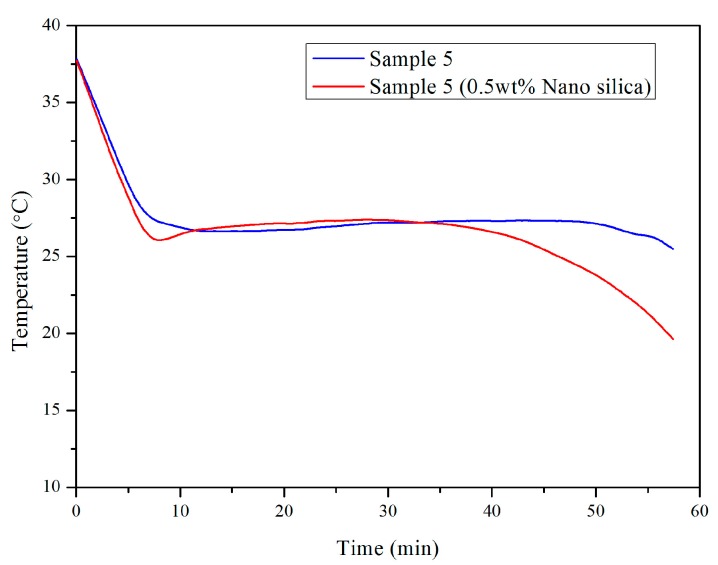
Cooling curve of Sample 5 with and without 0.5 wt % nano SiO_2_ particles.

Based on the better performance of the 0.5 wt % dosage of nano SiO_2_ particles in Sample 6, the same dosage was also utilized in Sample 5 so as to evaluate its effect in reducing supercooling. The cooling curves are presented in Figure 8. The cooling curve of Sample 5 composited with 0.5 wt % nano SiO_2_ particles is smoother than the sample without nano particles. After adding the nano particles, the supercooling degree of Sample 5 reduced from 1.5 °C to 0.4 °C. This shows that the supercooling of the SiO_2_-CaCl_2_·2H_2_O-CaCl_2_ salt hydrate PCM was almost eliminated. Hence, it can be deduced that during solidification, nano SiO_2_ particles have a positive effect on crystal growth. It can be inferred that CaCl_2_·2H_2_O, as an effective nucleation agent, can reduce the substantial supercooling of the PCM system, while nano SiO_2_ particles have a positive effect in reducing supercooling. With the synergistic action of the two materials, the performances and properties of the newly developed PCM system can be better—especially in terms of supercooling—than reported in previous research [7,14,22,23]. We would like to mention here that no segregation occurred for all of the PCMs that were used in this research.

**Figure 9:**
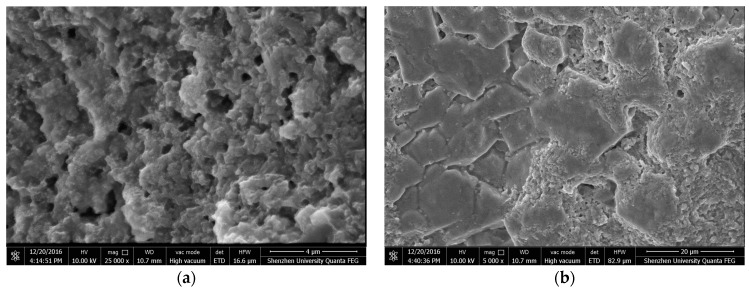
ESEM photographs of Sample 6 adding 0.5 wt % nano SiO_2_ particles (**a**) 25,000× magnification; (**b**) 5000× magnification.

**Figure 10:**
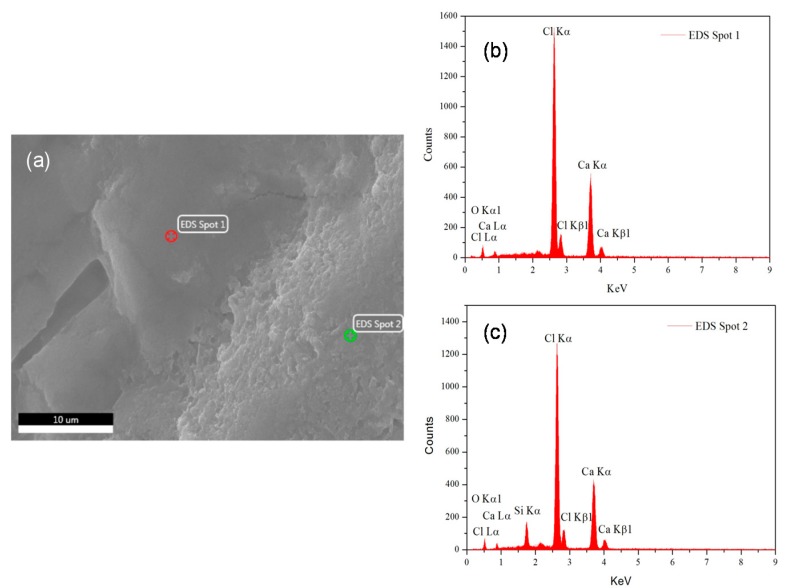
EDS (Energy Dispersive Spectrometer) photograph of Sample 6 with 0.5 wt % nano SiO_2_ particles (**a**) ESEM micrograph with two EDS spots; (**b**) EDS results of spot 1; (**c**) EDS results of spot 2.

The micrographs of Sample 6 containing 0.5 wt % nano SiO_2_ particles are shown in Figure 9. The crystal structure of the sample seems to be irregular. In fact, due to the synergetic effect of the salt hydrate PCM and the nano SiO_2_ particles, the PCM was closely bounded by nano SiO_2_ particles (Figure 9a). This shows that the crystallization of a PCM is affected by nano particles. Figure 9b shows the micrograph of the prepared composite PCM at a lower magnification. Although ultra-sonication is an effective method in dispersing nano SiO_2_ particles, the dispersion effect was not, however, perfect (Figure 9b). From the SEM photograph, and based on the peak intensity of element Si (Figure 10a–c), it can be inferred that the PCMs with a smooth surface have a lower concentration of nano SiO_2_ particles than the PCMs with a rough surface. This shows that ultra-sonication should be having an influence on the quality of the final product prepared by combining nano SiO_2_ particles and PCM, which, in turn, has a relationship with the supercooling’s reduction. Hence, the presence of too many smooth surface areas may result in the formation of nano SiO_2_ floccus, thereby reducing the efficiency of a composite PCM. It is therefore suggested that in the future, researchers should focus on parameters influencing the formation of these smooth surfaces.

## 4. Conclusions

In this research, a new method was used to prepare CaCl_2_·2H_2_O and CaCl_2_ as a novel salt hydrate PCM to reduce supercooling. The XRD results showed that the peak position of all of the prepared samples mainly matched with the standard XRD pattern of pure CaCl_2_·6H_2_O. Therefore, all of the samples can be considered to follow the phase-transition mechanism of CaCl_2_·6H_2_O. The solution was prepared by fast freezing the samples to −20 °C, which was found to be a successful method. With this new method, salt hydrate PCMs based on CaCl_2_·6H_2_O can be prepared rapidly.

Salt hydrates have issues of supercooling when they are utilized as PCM. The supercooling degree of the salt hydrate PCM reduced with the increase in volume of CaCl_2_·2H_2_O. The salt hydrate PCM containing 66.21 wt % CaCl_2_·2H_2_O reduced the supercooling degree by about 96.8%. Hence, CaCl_2_·2H_2_O could be recognized as nucleation agent, which reduced the supercooling of the PCM by a large margin, i.e., from 24.7 °C to 0.8 °C (98.8%). For the different dosages of CaCl_2_·2H_2_O tried in this research, the minimum volume acceptable for reducing the supercooling of the PCM should be greater than 34.03 wt %. All of the prepared samples showed relatively higher enthalpy (greater than 155.29 J/g), and have the possibility to be used for thermal energy storage purposes. Hence, CaCl_2_·2H_2_O plays an important role in reducing supercooling, and it can be helpful in adjusting the solidification enthalpy.

The prepared PCM samples incorporated with SiO_2_ (Sample 6 and 5) reduced the supercooling degree to 0.2 °C and 0.4 °C respectively. Hence, the hydrate salt PCM samples incorporated with SiO_2_ nearly eliminated supercooling, and have promising potential. The different cooling conditions used for preparing the PCM were found to be effective in reducing the supercooling degree of the PCM. Therefore, the novel composite PCMs can be used for thermal energy storage applications.

From the ESEM images, it was found that the ultra-sonication used to disperse nano SiO_2_ in the prepared PCM might impact the supercooling degree of the PCM. Hence, for better results (in terms of supercooling), it must be ensured that nano SiO_2_ are dispersed uniformly in salt hydrate PCMs.

## Figures and Tables

**Table 1:** Solubility of calcium chloride in water (100 g) at different temperatures.

Temperature (°C)	0	10	20	30	40	60	80	100
Solubility (g)	59.5	64.7	74.5	100	128	137	147	159

**Table 2:** Salt hydrate phase change materials (PCMs) with different CaCl_2_·2H_2_O and CaCl_2_.

Parameters and Items	Sample No.					
	1	2	3	4	5	6
*x* (g)	50.00	39.58	29.17	18.75	8.33	0.00
*y* (g)	0.00	20.42	40.83	61.25	81.67	98.00
*z* (g)	50.00	50.00	50.00	50.00	50.00	50.00
*x* + *y* (g)	50.00	60.00	70.00	80.00	90.00	98.00
*y*/(*x* + *y*) (%)	0	34.03	58.32	76.56	90.74	100
*x*/(*x* + *y* + *z*) (%)	50.00	35.98	24.31	14.42	5.95	0
*y*/(*x* + *y* + *z*) (%)	0	18.56	34.03	47.12	58.34	66.21

Notes: *x*: mass of CaCl_2_; *y*: mass of CaCl_2_·2H_2_O; *z*: mass of H_2_O.

**Table 3:** Thermal properties of all samples evaluated by DSC.

Items	Sample 1		Sample 2		Sample 3		Sample 4		Sample 5		Sample 6	
	Mean	Standard Deviation	Mean	Standard Deviation	Mean	Standard Deviation	Mean	Standard Deviation	Mean	Standard Deviation	Mean	Standard Deviation
Mass of samples (mg)	12.0	0.2	17.9	0.2	27.6	0.4	17.8	0.2	23.8	0.3	14.5	0.3
T_eo_ (°C)	27.1	0.3	28.1	0.3	27.9	0.4	27.8	0.5	27.4	0.4	27.4	0.3
T_m_ (°C)	33.7	0.5	36.2	0.4	38.6	0.5	35.3	0.3	35.6	04	33.1	0.4
H (J/g)	205.13	2.1	181.02	1.8	174.37	1.9	178.60	1.6	167.44	1.9	155.29	1.6

Notes: T_eo_: start phase-transition temperature; T_m_: complete melting temperature; H: solidification enthalpy.

**Table 4:** Reduction in supercooling degree with the addition of SiO_2_ nanofluids.

Additive Volume (%)	T_m_ (°C)		T_n_ (°C)		ΔT (°C)	
	Mean	Standard Deviation	Mean	Standard Deviation	Mean	Standard Deviation
0	26.6	0.4	25.7	0.6	0.8	0.2
0.1	26.0	0.3	25.1	0.4	0.9	0.2
0.3	27.6	0.4	27.3	0.3	0.3	0.1
0.5	27.3	0.5	27.1	0.5	0.2	0.1

Notes: T_m_: phase change temperature; T_n_: supercooling temperature; ΔT: supercooling degree.
